# A simulation model of the within-host dynamics of *Plasmodium vivax* infection

**DOI:** 10.1186/s12936-015-0580-z

**Published:** 2015-02-05

**Authors:** Douglas H Kerlin, Michelle L Gatton

**Affiliations:** Infectious Diseases Programme, QIMR Berghofer Medical Research Institute, Brisbane, QLD Australia; School of Public Health and Social Work, Queensland University of Technology, GPO Box 2434, Brisbane, QLD 4001 Australia

**Keywords:** *Plasmodium vivax*, Simulation model, Preferential invasion, Hypnozoite, Within-host dynamics

## Abstract

**Background:**

The benign reputation of *Plasmodium vivax* is at odds with the burden and severity of the disease. This reputation, combined with restricted *in vitro* techniques, has slowed efforts to gain an understanding of the parasite biology and interaction with its human host.

**Methods:**

A simulation model of the within-host dynamics of *P. vivax* infection is described, incorporating distinctive characteristics of the parasite such as the preferential invasion of reticulocytes and hypnozoite production. The developed model is fitted using digitized time-series’ from historic neurosyphilis studies, and subsequently validated against summary statistics from a larger study of the same population. The Chesson relapse pattern was used to demonstrate the impact of released hypnozoites.

**Results:**

The typical pattern for dynamics of the parasite population is a rapid exponential increase in the first 10 days, followed by a gradual decline. Gametocyte counts follow a similar trend, but are approximately two orders of magnitude lower. The model predicts that, on average, an infected naïve host in the absence of treatment becomes infectious 7.9 days post patency and is infectious for a mean of 34.4 days. In the absence of treatment, the effect of hypnozoite release was not apparent as newly released parasites were obscured by the existing infection.

**Conclusions:**

The results from the model provides useful insights into the dynamics of *P. vivax* infection in human hosts, in particular the timing of host infectiousness and the role of the hypnozoite in perpetuating infection.

## Background

Historically, the high profile and lethal reputation of *Plasmodium falciparum* has meant that the bulk of malaria research and management has focused on this parasite; comparatively, the importance of *Plasmodium vivax* (and other species of human malaria) as a human pathogen has been neglected [1,2]. Yet despite a preconception that *P. vivax* is a benign infection, at least in comparison with *P. falciparum*, vivax malaria is a significant burden to nearly 40% of the world’s population, with estimates of between 132 and 391 million cases per year [2]; the greatest burden (52%) observed is in South and East Asia. Multiple reports have linked infection with *P. vivax* to patient death, often as a result of fatal complications and particularly in pregnant women and young children [3,4].

As the preconception of *P. vivax* as a benign disease agent is challenged, key questions remain regarding the basic ecology and dynamics of *P. vivax* infection [2].

The capacity of researchers to address these questions has been hampered by ongoing difficulties in producing an *ex vitro* culture system. Such a system was developed for *P. falciparum* in the 1970s [5] and drove subsequent research which provided a wealth of information on aspects of the parasite and infection [6]. With limited opportunities to investigate the dynamics of the parasite in the laboratory, modelling provides a useful alternative to explore aspects of *P. vivax* infection.

This manuscript describes the development of a stochastic simulation model of the within-host dynamics of a *P. vivax* infection in a naïve host, enabling elucidation of a number of significant questions regarding the interaction between host and parasite. It is intended that the development of this within-host model will subsequently have utility as the basis for a between-host model which incorporates multiple hosts and vectors. As *P. falciparum* is far better studied than *P. vivax*, the dynamics of this parasite is used as the basis for the model. However, it is important to recognize that there are several significant features that differentiate *P. vivax* from *P. falciparum.* These are detailed below.

### Preferential red blood cell invasion

*Plasmodium falciparum*, while showing a weak preference for younger red blood cells (RBCs), is generally assumed to be capable of invading all RBC age classes, whilst *P. vivax* shows a strong preference for the youngest RBCs, reticulocytes [7,8]. It has been hypothesized that preferential invasion may play a key role in regulating the abundance of parasites in vivax malaria; increased competition between merozoites for a limited population of susceptible RBCs can slow the growth of an infection, ensuring that the host remains alive sufficiently long to maximize the chances of subsequent generations reinfecting a feeding mosquito [9]. Certainly the less discriminant *P. falciparum* is the human malaria species predominantly responsible for cases of severe disease and mortalities, while the other human Plasmodium species, which are more discerning in the RBCs they invade, are considered to be more benign disease agents [10].

### Pyrogenic thresholds

Fever is the most common clinical manifestation of malaria infection, and is a primary mechanism by which the host immune system can control infection, with increased body temperatures associated with parasite death. Higher fevers generally lead to greater parasite mortality. Fever is triggered by the rupture of mature schizont stage parasites and associated RBC lysis, with the pyrogenic threshold representing the density of parasites required to stimulate a febrile response to infection. Vivax malaria is generally accepted to have a lower pyrogenic threshold than falciparum malaria [2,11], thus fewer *P. vivax* parasites are required to trigger a fever in the host.

### Production of gametocytes

During the erythrocytic stage of parasite development in humans, a fraction of parasites commit to a sexual development pathway. Different cells will produce male and female gametocytes, which can then be transmitted to a mosquito during a blood meal. *Plasmodium vivax* gametocytes vary from their *P. falciparum* equivalents by their presence in the blood at detectable levels during the primary attack of the disease whereas *P. falciparum* gametocytes cannot usually be seen on microscopy until some 9–12 days post infection [12]. It is unclear whether this difference is due to *P. falciparum* gametocytes sequestering in the bone marrow, or a longer gametocyte development time. The survival time of *P. vivax* gametocytes also appears to be significantly shorter than that of *P. falciparum* gametocytes [12].

### Antigenic variation

Approximately 12–14 hours after invasion, RBCs parasitized by *P. falciparum* express a strain-specific variant surface antigen (*P. falciparum* erythrocyte membrane protein 1 *Pf*EMP1), which mediates agglutination and adhesion of infected erythrocytes to the vascular endothelium and results in parasitized RBCs sequestering in the deep tissues, protected from splenic destruction [13,14]. PfEMP1 is encoded by a highly diverse family of genes, known as *var* genes, enabling rapid switching of presented antigens to evade the host immune response. While PfEMP1 and *var* gene switching have been the focus of intense study in *P. falciparum*, no clinically relevant equivalent has been identified in *P. vivax* [15].

### Hypnozoites

Hypnozoites, an exo-erythrocytic parasite life cycle stage derived from the initial inoculum of sporozoites, are characteristic of *P. vivax*, but not *P. falciparum* [16,17]. The hypnozoite stage is believed to provide a mechanism for the parasite to survive in environments where the presence of the mosquito vector is intermittent (e.g. in Finland or the Korean Peninsula, where mosquitoes overwinter during the winter months [18,19]). It is generally assumed that the duration of this dormant stage is genetically determined, but it has also been hypothesized that mosquito bites, or a host immune response triggered by other infections may ‘alert’ the hypnozoite to the presence of the vector [20].

## Methods

The basis of this work is a previously reported simulation model of preferential invasion of *Plasmodia* merozoites [9]. In brief, the probability of merozoites successfully invading a new host red blood cell was assumed to be a function of the relative abundance of RBCs (anaemic status of the host or how likely is it that a free merozoite will encounter an RBC) and the relative abundance of susceptible RBC age classes (having encountered an RBC, how likely is it that the RBC is of a susceptible age class). As an infection progresses, the probability of a free merozoite encountering a susceptible RBC is expected to fall. Such a fall reduces the probability of a successful merozoite invasion. As it is widely recognized that *P. vivax* preferentially invades reticulocytes, merozoite invasion was restricted to 1 and 2 day old RBCs.

Mathematically, the number of merozoites which successfully invade RBCs is determined by drawing from the Binomial distribution $B\left({\mathit{\mathsf{N}}}_{\mathit{\mathsf{s}\mathsf{chizont}}{\mathit{\mathsf{s}}}_{\mathit{\mathsf{i}}}}\times {\mathit{\mathsf{m}}}_{\mathit{\mathsf{m}\mathsf{ax}}},{\mathit{\mathsf{p}}}_{\mathit{\mathsf{i}}}\right)$, where ${N}_{schizont{s}_i}$ is the number of schizont stage parasites rupturing on day *i* and releasing merozoites, *m*_*max*_ is the maximum potential number of merozoites released per schizont, and *p*_*i*_ is the probability of successful invasion on day *i*:

$$ {p}_i=\frac{N_{tota{l}_i}}{N_{tota{l}_o}}\times \frac{N_{sus{c}_i}}{N_{sus{c}_o}}\times {c}_i\times {0.7}_{,} $$

where $\frac{N_{tota{l}_i}}{N_{tota{l}_o}}$ is representative of relative RBC density (the degree of anaemia in the host) and $\frac{N_{sus{c}_i}}{N_{sus{c}_o}}$ the proportion of susceptible RBCs available in the host on day *i* relative to day *0*, while *c*_*i*_ is a time-dependent clonal immunity function and 0.7 a scaling factor providing an adjustment from the biological maximum number of merozoites released (*m*_*max*_), to the actual mean number of merozoites which usually develop and invade a new RBC in naïve hosts. It is assumed that ${N}_{tota{l}_o}$ is 2.5 × 10^13^ and that ${N}_{sus{c}_o}$ is approximately 4.17 × 10^11^ (${N}_{sus{c}_o}$ is approximately equal to ${N}_{tota{l}_o}$/60, but the exact value is dependent on the stochastic distribution of RBCs to the various age classes). The dynamics of the RBC (ie ${N}_{tota{l}_i}$ and ${N}_{sus{c}_i}$) are as previously reported [9]. The immunity function is calculated as follows: ${c}_i=\beta +\left(1-\beta \right)\times {e}^{\frac{\hbox{-} \mathrm{i}}{10}}$, where *β* is drawn from a gamma distribution fitted during the modelling process. The only change to this model from the previous study [9] was that the duration over which clonal immunity develops was halved, based on declines in parasitaemia over time from published patient records [21]. It is assumed *m*_*max*_ is equal to 18 [8], resulting in an average of 12.72 released merozoites per replication cycle (Q_1_ = 11, Q_3_ = 14) at the start of an infection (*i* = 0).

If the number of successful merozoites exceeds the number of available susceptible RBCs, the excess are removed from the system.

To develop the model of within-host dynamics this base model was augmented to incorporate the effects of fever, the production of gametocytes and the release of dormant hypnozoite stages of the parasite. These additions were created as separate modules. In the absence of an identifiable mechanism of clinically relevant antigenic variation, it is assumed that the clonal immunity described above is the only specific host immune response that develops during infection. The model uses a 24 hour (1 day) time step and the duration of the erythrocytic cycle in *P. vivax* was assumed to be 48 hours; variability in the duration of the erythrocytic cycle and differences in the duration of each stage of the erythrocytic lifecycle [22] were not considered.

### Fever

Representation of the fever response is largely derived from the work of Gatton and Cheng on *P. falciparum* [23], with some changes to account for known differences with *P. vivax*. Pyrogenic threshold figures for *P. vivax* have been reported in a number of prior studies; 310 parasites/μL in southern Papua, Indonesia [24], 500 parasites/μL in analyses of children in Papua New Guinea [25], and 181 parasites/μL in Thailand [26]. For the current model, a pyrogenic threshold of 300 ± 200 parasites/μL is adopted. This value is considerably lower than the comparative value of 1,644 - 2,500 parasites/μL for *P. falciparum* [11,27]. Since information on the dynamics of the threshold during an infection is missing for *P. vivax*, it is assumed this threshold is not reached for a period of 7 days, and introduce a log-linear increase over these first seven days, following work by Gatton and Cheng on the pyrogenic threshold of *P. falciparum* [27].

When parasitaemia exceeds the pyrogenic threshold, a fever response is triggered. The strength of this response (*f*_*i*_) is equal to the number of additional parasites exceeding the threshold, as a proportion of the threshold, multiplied by a stochastic variable representative of the ‘strength’ of the fever response (*g*_*i*_):

$$ {f}_i=\left\{\begin{array}{c}\hfill 0,\hfill \\ {}\hfill {g}_i\frac{N_{par{a}_i}}{N_{threshol{d}_i}},\hfill \\ {}\hfill 10\hfill \end{array}\right. $$

where ${N}_{par{a}_i}$ is the number of parasites on day *i* and ${N}_{threshol{d}_i}$ is the fever threshold as previously defined. *g*_*i*_ is modelled using a gamma distribution, fitted during the modelling process. *f*_*i*_ is limited to a minimum value of 0, and a maximum value of 10. The proportion of parasites surviving fever is assumed equivalent to *exp*^*-αf*^. Different parasite life cycle stages exhibit different susceptibilities to fever; the *α* term is used to incorporate this into the model.

Following a review of the relevant literature [28,29], the model allows fever to impact on parasite survival such that late stage parasites are more susceptible to fever than early stage parasites (remembering that fever is most contemporaneous with early ring stage parasites, occurring shortly after schizont rupture), while gametocytes are considered less susceptible than ring stage parasites. Within the gametocyte sub-population, late stage gametocytes are considered more susceptible to fever than early stage gametocytes. Thus *α* = 1 for non-rupturing (late stage) asexual parasites, *α* = 2 for rupturing asexual parasites, *α* = 0.364 for early-stage gametocytes, and *α* = 0.667 for late-stage gametocytes.

### Gametocytes

It is assumed that gametocyte production begins at the onset of infection and that the gametocyte conversion rate (GCR) – the rate at which merozoites commit to the gametocyte development pathway – is log-normally distributed. A mean of the natural logarithm of GCR equal to −1.59, with a standard deviation of 0.13, is used based on published GCRs of 1.4% to 4.7% (mean of 3%) [30] and 4% [31]. There is also considerable evidence that the gametocyte conversion rate is elevated under stressful conditions that are unfavourable for parasite survival [12]. To incorporate this into the model, the GCR was allowed to rise by 2% on average when fever is present, following a previous model of gametocyte conversion in *P. falciparum* [27]. All gametocytes are assumed to require two days to mature before they became infectious to mosquitoes [32,33], and all survived for a further three days thereafter (with no stochastic variation) [34,35]. This longevity is considerably shorter than reported values for *P. falciparum*, however the reduced longevity of *P. vivax* gametocytes is offset by a higher rate of production [12].

Given the desire to develop a model that can be subsequently augmented with a between-host model of transmission, the predicted the number of gametocytes required to ensure at least one male and one female is taken up with any mosquito blood meal was also considered. The sex ratio of *P. vivax* gametocytes is difficult to ascertain in the absence of a reliable culture system. To predict the density of gametocytes required to ensure the carriage of at least one male and one female in a mosquito blood meal, a generic sex ratio for malaria is used - one male gametocyte is assumed to be created for every 10 female gametocytes [28]. Using a negative binomial distribution where the probability of finding a male gametocyte (*p)* is thus equal to 0.09, the predicted the number of gametocytes required to ensure at least one male and one female with 95% confidence is 31. Two assumptions are made in order to calculate this value: 1) a feeding mosquito will take a blood meal equivalent to 1 μL of blood [36], and 2) the distribution of gametocytes ingested by a feeding mosquito follows a negative binomial distribution with constant overdispersion, where *k* = 3.105 [37].

### Hypnozoites

At the onset of infection, it is assumed that between one and four hypnozoites develop within the liver, each with a pre-programmed relapse date. This assumption was made on the basis of observations of infected hosts demonstrating recurrent relapses in the absence of reinfection; it is uncertain whether this repeated recurrence is due to multiple hypnozoites with different relapse dates developing during the initial infection, or new hypnozoites being created during each subsequent relapse event. Detailed data was available describing relapse patterns in three different regions, allowing the generation of three different relapse scenarios. For the purposes of this study the focus is on a *Chesson* style relapse pattern. Chloroquine studies from New Guinea and the Pacific describe a rapid pattern of relapse, with a duration of sequestration modelled using a combination of normal (87% - *μ* = 22.7, *σ* = 2.14) and binomial (13% - *N* = 30, *p* = 0.95) distributions (given a degree of bimodality in the source data), and providing a mean time between primary infection and first relapse, and between subsequent relapses, of 23.8 days [38]. This short relapse time falls within the mean time to relapse predicted for parasites in the wider South-East Asia region [39]. While the Chesson strain is largely confined to South-East Asia, this rapid pattern of relapse has resulted in the Chesson strain of *P. vivax* gaining a central role in experimental studies of the species [40].

It is assumed that in 40% of new infections only one hypnozoite is sequestered. A pre-programmed relapse time is assigned by a random draw from the probability distribution described above and converted to a relapse date. Two, three or four hypnozoites are sequestered in 30%, 20% and 10% of infections, respectively; where multiple hypnozoites are sequestered, each is also assigned a relapse date by a random draw, multiplied by the hypnozoite number (e.g. a second hypnozoite, randomly assigned a relapse time of 25 days, will be assigned a relapse date of 25 × 2 = 50 days after the new infection is created).

Once the relapse date is reached, 10,000 merozoites are released from the hypnozoite. These merozoites are treated in the same way as a new infection in the model.

### Model implementation

The overall within-host model was implemented as a series of decision steps. Day 0 represented the release of 10,000 merozoites in the primary infection. For each subsequent day of the simulation:

1. Check to determine if fever will be triggered by the rupturing RBCs,
2. If schizonts have matured, or hypnozoites have reached their relapse date then merozoites are released from within rupturing schizonts or from relapsing hypnozoites, or both:
  - Merozoites compete to find a suitable host cell according to the preferential invasion module,
  - A fraction of the merozoites commit to the gametocyte pathway,
3. Any existing gametocytes age one day and gametocyte death occurs if necessary,
4. If fever has been triggered, parasite death is simulated with a proportion of the parasite burden removed from the each parasite age class.

### Fitting the model

The model was fit using digitized copies of six published time series of parasitaemia, taken from neurosyphilis patients treated with *P. vivax* infection [21]. Time series run for a maximum of 73 days before treatment, so the model simulation period was 80 days. Two variables in the model were not informed by the literature, and were instead fitted using available data. These variables were the strength of the clonal immunity response (*β*), and strength of the ‘killing’ power of fever (*g*). Gamma distributions were used to model each variable. After aligning the initial growth of the infection to account for the delay between exo-erythrocytic schizont rupture and patent parasitaemia, simulated annealing was used to fit the model to the mean of the six available time-series.

The fitted model was subsequently used to simulate a set of 1,000 infections, each within a malaria-naïve host, which were used to determine 95% confidence intervals for model output. These numbers were then compared to a more comprehensive set of summary statistics reported for 48 sporozoite induced *P. vivax* infections in the same neurosyphilis study (henceforth referred to as the ‘McKenzie summary statistics’, or MSS) to validate the model: peak parasitaemia, day of peak parasitaemia, day of peak fever, peak gametocytes and day of peak gametocytes. Statistics recorded for trophozoite-induced infections, while available, were not considered, as the current model was based on a single release of merozoites from a single hepatic schizont, and thus more closely resembled a sporozoite-induced infection.

A version of the fitted model was also run where chemotherapy was used to terminate infections. This model was included to clearly demonstrate the utility of the hypnozoite release component of the model. The treatment was assumed to kill all asexual parasites and gametocytes, and have no residual activity.

All analyses were conducted in the R statistical computing environment [41].

## Results

**Figure 1:**
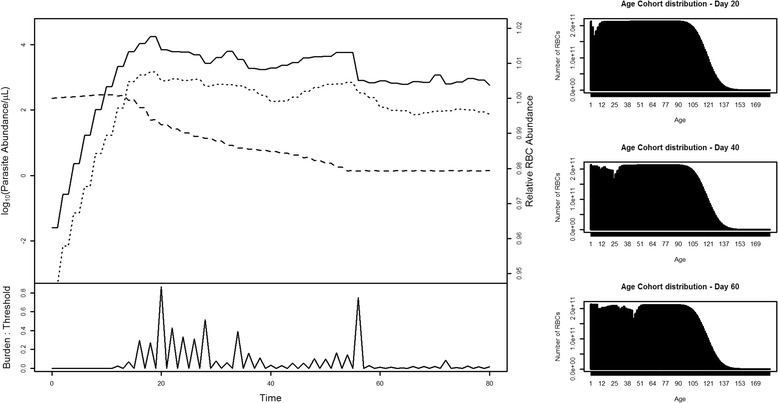
**Example time course of a modelled infection.** The infection commences with the release of 10,000 merozoites on Day 0. Subsequent changes in log(parasite) (solid line), log(gametocyte) (dotted line) and host RBC relative abundance (dashed line) are illustrated in the top-left panel. The lower left panel shows the ratio of parasite burden to fever threshold. Histograms to the right show the age profile of the host RBC population (and thus the impact of preferential invasion on the RBC age profile) at three points (Day 20, 40 and 60) during the infection.

**Figure 2:**
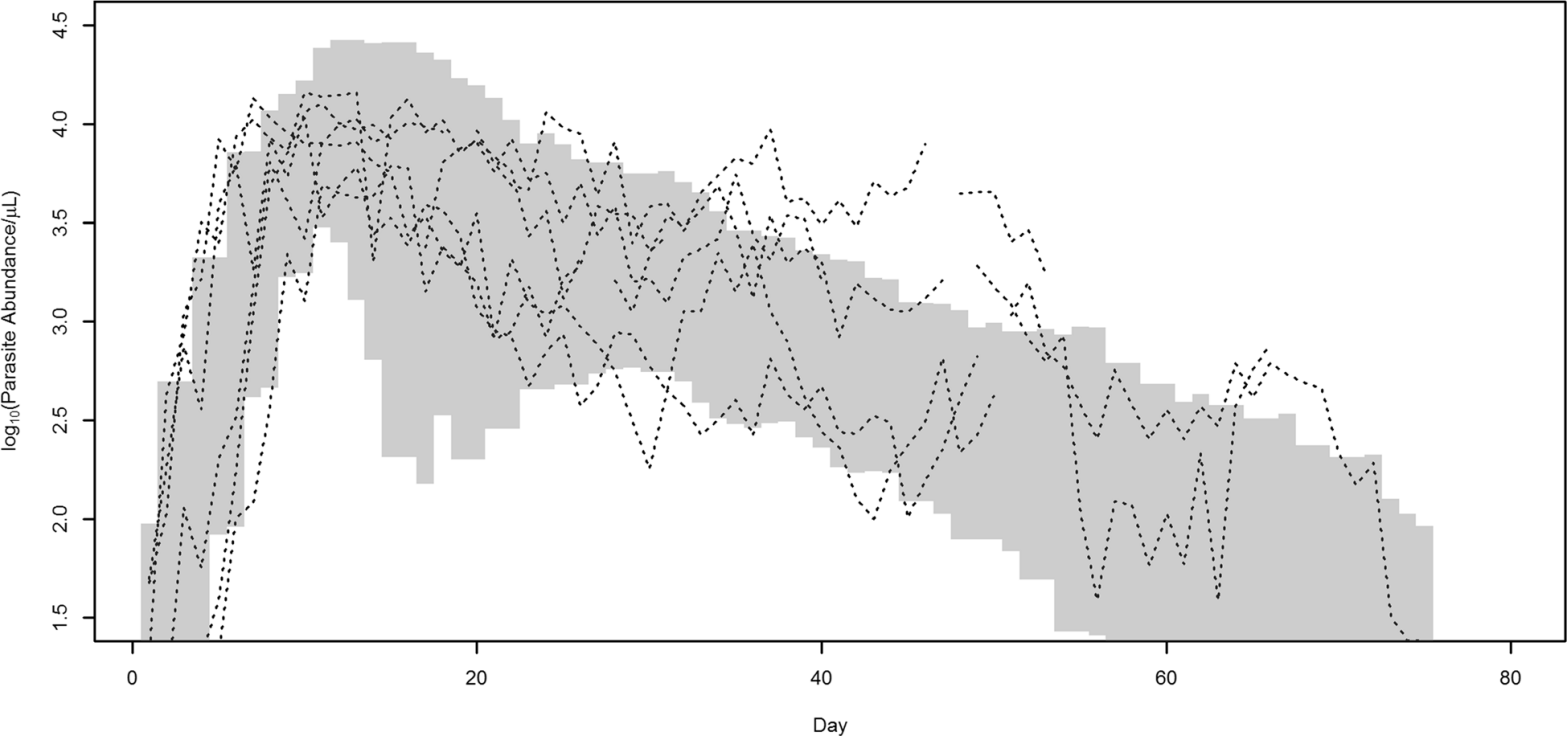
**95% confidence intervals for simulated** ***P. vivax*** **log** ***(*** **parasitaemia) produced by the fitted model.** Dotted lines represent the six digitized time series (drawn from McKenzie *et. al.* [21]) used to fit the model.

Sample output for a single iteration of the fitted model is presented in Figure 1. The two parameters fit using the data were estimated as: *β* = Gamma(9.433, 193,134), *g* = Gamma(2.317, 160.083). Following 1,000 simulations of the fitted model, confidence intervals around parasitaemia were developed (Figure 2). Steps in the initial amplification of parasitaemia reflect the tertian nature of the infection; replication only occurs every second day. The typical pattern for dynamics of the parasite population generally is a rapid exponential increase in the first ~10 days, followed by a more gradual decline to day 80. Gametocyte counts follow a similar trend, but approximately two orders of magnitude lower. Mean days to patent parasitaemia was estimated to be six days post release of blood-stage parasites from the liver (95% confidence intervals 3.6-8.4 days).

After simulating 1,000 infections the summary statistics for comparison with the MSS were calculated (Table 1). Model estimates generally display similar timings of key events (peak parasitaemia, peak gametocytes) when compared to MSS figures. However, estimates of density were generally much larger than reported in MSS.

**Table 1:** **Comparisons between the model output and summary statistics for 48 sporozoite-induced infections (drawn from McKenzie** ***et. al.*** [21]**)**

	**Min**	**Max**	**Median**	**Mean**	**Std dev**
Day of 1st parasite peak	14 (5)	32 (34)	18 (10)	19.4 (11.4)	3.0 (5.9)
Peak parasitaemia (/μL)	5567 (693)	53246 (20376)	14939 (8131)	16475 (8675)	6384 (4964)
Day of 1st fever	16 (3)	36 (22)	20 (8)	21.7 (NA)	3.6 (4.1)
Peak fever	0.447	8.336	0.983	1.134	0.574
Day of peak gametocytes	16 (7.5)	38 (29.5)	20 (17)	21 (18.1)	3.8 (6.0)
Peak gametocytemia (/μL)	479 (20)	4939 (840)	1294 (152)	1415 (209)	548 (NA)

Values reported by McKenzie *et al.* are listed in parentheses where available. Peak fever represents the proportion of parasites in excess of the fever threshold; no equivalent measurements were available from McKenzie *et. al.* [21]. For the model outputs, Day 0 represents the day the exo-erythrocytic schizont releases merozoites into the host circulation.

The period of host infectivity can be determined by analysis of the density of gametocytes in the host. It is estimated that a density of 31 gametocytes/μL of host blood will ensure at least one male with 95% confidence. It is assumed that due to the skewed sex ratio in favour of females that the likelihood of a 1 μL sample containing 31 males (no females) is trivial in comparison to the likelihood of 31 females (no males). Given aggregation in the distribution of gametocytes in host blood, a density of 116.1 gametocytes/μL is required to ensure with 95% confidence that any particular blood meal of 1 μL will contain at least 31 gametocytes, and thus at least one male and one female gametocyte. The model predicts that, on average, an infected naïve host in the absence of treatment will exhibit gametocytes densities greater than 116.1/μL for a mean of 34.4 days (95% CI 22, 46 days), first reaching this threshold on average 7.9 days post patency (95% CI 5, 10).

**Figure 3:**
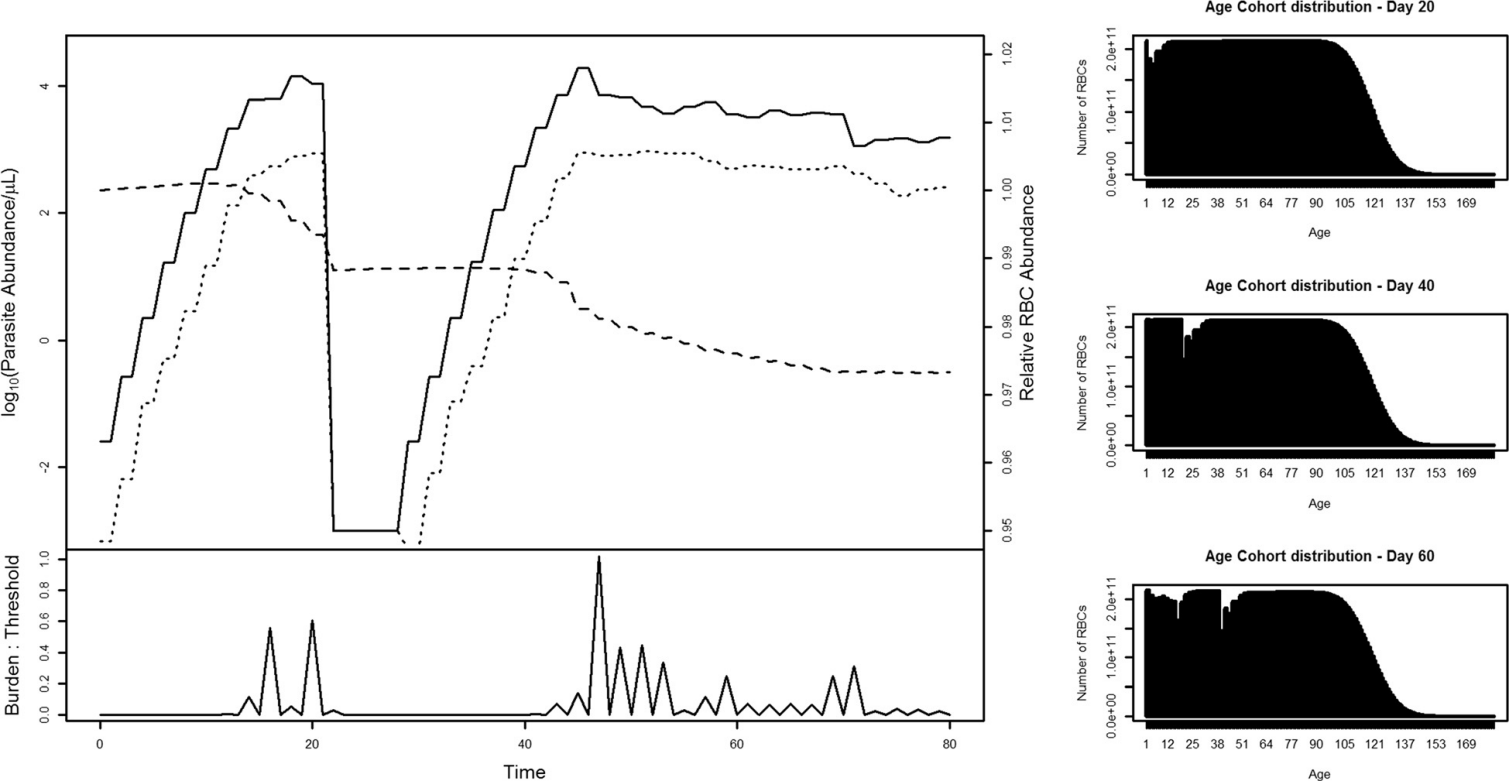
**Model demonstration of hypnozoite-mediated relapse following chemotherapy intervention.** The initial infection is cleared by treatment on day 20 prior to relapse on day 25.

Hypnozoite-mediated relapse was modelled throughout (Figure 1). However, in the absence of any treatment to remove the existing parasite population, the magnitude of the original parasite population obscures the new relapse induced infection, preventing detection of this second, hypnozoite-induced peak. Indeed, in some simulations, fever associated with the initial infection was of sufficient severity to kill the relapse parasites before they became established. To graphically demonstrate the relapse feature in the model, a trivial drug treatment module was incorporated to allow for the removal of the initial infection, and permit the relapsing infection to grow in the absence of this fever (Figure 3).

## Discussion

A model of the infection dynamics of *P. vivax* in a naïve host has been developed. This model is founded on a model of preferential merozoite invasion incorporating non-variant clonal immunity that captures the high affinity displayed by *P. vivax* merozoites for reticulocytes, and the impact this may have on limiting parasite population growth. The model, augmented by the inclusion of fever, the production of gametocytes, and hypnozoite-mediated relapse, can be used to answer questions regarding the infectivity of hosts and offer suggestions as to the role of hypnozoite-mediated relapse in the evolution of drug resistance.

The model presented is a simple abstraction of a true infection. The model was fit to a small subset of data from neurosyphillis patients as no other time-course data was readily available. Comparisons between model output and available data thus require some caveats. For example, it is unrealistic to expect a perfect match between the model and the summary data: it is not clear how representative the six available time-series are of the neurosyphilis dataset as a whole, there is no way to quantify observation error in the summary data, and timings are dependent on the fitting process. The similarity between model output and the summary MSS data does however suggest that the six available time-series do provide a reasonable representation of the larger data set. Thus it is not surprising that the model is unable to predict the full variation present in patient data. It may be that fitting to the range of values for the six time series, rather than simply the mean, or adding additional parameters would improve model fit, as would having more than six time series to use for fitting. Despite this shortcoming, the model does appear to provide a reasonable description of the infection dynamics, and can subsequently still provide some useful insights into the biology of *P. vivax*.

The timing of the various peaks of infection are generally earlier in the MSS than in the model output, though in some instances (e.g. maximum day of first parasite peak, median/mean day of peak gametocytes) the estimates are similar. Some part of this is a consequence of the fitting process, but generally the cause appears to be the slow decay in the MSS following the peak. The model had difficulty replicating this decline, and has likely compensated by overshooting the peak, allowing a sharper decline to more closely match the MSS dataset. Thus, the model has overestimated the peak parasitaemia, in order to better fit the slower decline following the peak.

It is unsurprising that estimates of density were generally much larger in the model output than reported in MSS; MSS statistics are a product of microscopy of patient blood, which is subject to the transient nature of parasite detection in peripheral blood. Factors such as parasite sequestration and non-synchronized rupture of parasites impact on the parasite density observed. These factors are not accounted for in the model, therefore it is expected that the model would over-estimate the measured density.

One of the objections for preferential invasion as a mechanism for regulating parasite abundance is that if parasitaemia is suppressed, it follows that gametocyte abundance is also suppressed, reducing the probability of transmission to a new host. The model presented offers evidence that refutes this objection, demonstrating the possibility that preferential invasion can regulate parasite abundance while producing sufficient gametocytes to ensure an infectious host for a period of more than a month. It is further worth noting that the figures quoted here assume a worst case scenario of 1 male gametocyte produced for every 10 female gametocytes, when the generic sex ratio for malaria parasites suggest the proportion of male gametocytes could be as high as 1 in 5, greatly reducing the parasite density required to make a host infectious.

Hypnozoite-mediated relapse provides *P. vivax* infections a sequestering mechanism, a potential sanctuary where the parasite can effectively lie dormant and await potentially more advantageous circumstances. At this stage it is not definitively known whether the hypnozoite is responsive to signals indicating a beneficial environment to re-activate an infection, or whether the parasite relies on chance, programming an ‘alarm clock’ to promote reactivation at a future time. The most notable conclusion to be drawn from the presented model is that hypnozoite-mediated relapse can be greatly suppressed by an existing infection and associated immune response. It is only when the host immune response, or treatment, sufficiently reduce the pre-existing infection that a hypnozoite-mediated relapse can be detected. Parasites from hypnozoites released prior to this time are absorbed into the existing parasite pool.

Given the short time span of available patient parasitaemia simulated by the model (maximum of 80 days), the relapse pattern typical of the Chesson strain of vivax malaria, a relapse pattern that is extremely short compared to others observed, was demonstrated. If a longer relapse pattern, such as demonstrated in strains from East Timor [42] or Sri Lanka [43], were used in the model, the probability that a relapsing infection would be suppressed by an existing infection greatly diminishes due to the reduction in frequency of fever throughout an infection. This raises an interesting question: if hypnozoites evolved in response to environmental conditions where the presence of the mosquito vector is intermittent, what is the purpose of a short relapse pattern such as exhibited by the Chesson strain? The original infection is likely to still be active, and mosquito abundance is unlikely to significantly change in such a short time period. This interesting evolutionary question warrants further consideration.

## Conclusions

In the absence of an *ex vitro* culture system for *P. vivax*, modelling provides a useful tool for exploring aspects of the biology and ecology of the parasite. Here a model of the dynamics of an infection within a host is presented and used to address questions about host infectivity, the role of preferential invasion in suppressing gametocyte production, and hypnozoite-mediated relapse. While such a model is ultimately an abstraction of a real infection, useful insights can be drawn from the outputs. Future work will allow the development of community-scale, multiple-host models, where parasite infections can be transmitted between hosts by mosquito vectors; such models will facilitate investigations into the suitability and effectiveness of interventions to control disease outbreaks and elimination/eradication strategies for *P. vivax*.
